# The Effect of Aligned and Random Electrospun Fibers Derived from Porcine Decellularized ECM on Mesenchymal Stem Cell-Based Treatments for Spinal Cord Injury

**DOI:** 10.3390/bioengineering11080772

**Published:** 2024-07-31

**Authors:** Zhiqiang Tai, Jiashang Liu, Bixue Wang, Shu Chen, Changsheng Liu, Xi Chen

**Affiliations:** Key Laboratory for Ultrafine Materials of Ministry of Education, Frontiers Science Center for Materiobiology and Dynamic Chemistry, Engineering Research Center for Biomaterials of Ministry of Education, School of Materials Science and Engineering, East China University of Science and Technology, Shanghai 200237, China; accounttzq@163.com (Z.T.); liucs@ecust.edu.cn (C.L.)

**Keywords:** decellularized extracellular matrix, aligned scaffolds, MSCs, spinal cord injury, nerve regeneration, electrospinning

## Abstract

The impact of traumatic spinal cord injury (SCI) can be extremely devastating, as it often results in the disruption of neural tissues, impeding the regenerative capacity of the central nervous system. However, recent research has demonstrated that mesenchymal stem cells (MSCs) possess the capacity for multi-differentiation and have a proven track record of safety in clinical applications, thus rendering them effective in facilitating the repair of spinal cord injuries. It is urgent to develop an aligned scaffold that can effectively load MSCs for promoting cell aligned proliferation and differentiation. In this study, we prepared an aligned nanofiber scaffold using the porcine decellularized spinal cord matrix (DSC) to induce MSCs differentiation for spinal cord injury. The decellularization method removed 87% of the immune components while retaining crucial proteins in DSC. The electrospinning technique was employed to fabricate an aligned nanofiber scaffold possessing biocompatibility and a diameter of 720 nm. In in vitro and in vivo experiments, the aligned nanofiber scaffold induces the aligned growth of MSCs and promotes their differentiation into neurons, leading to tissue regeneration and nerve repair after spinal cord injury. The approach exhibits promising potential for the future development of nerve regeneration scaffolds for spinal cord injury treatment.

## 1. Introduction

Traumatic spinal cord injury (SCI) is a destructive neurological disorder in which patients lose sensory and motor functions due to the destruction of long axons in the white matter tracts. This disruption presents significant challenges to the restoration of functions, positioning SCI as a formidable obstacle in the field of neurological recovery and rehabilitation [1,2]. In 2019, 20.6 million people worldwide suffered from spinal cord injuries [3], and the surging number of patients and high cost of treatment have triggered increasingly severe social and medical problems [4,5]. The pathophysiology of spinal cord injury (SCI) is often characterized by the degeneration of spinal cord tissue and subsequent neuronal loss, resulting in the creation of an unfavorable microenvironment that hinders nerve regeneration. The unfavorable microenvironment is marked by several pathological events, including the presence of inflammation, the development of spinal cord cavities, and the occurrence of glial scarring. The current clinical treatments for spinal cord injury (SCI) primarily involve a combination of surgical and pharmacological interventions, aiming to facilitate neurological function recovery in SCI patients while minimizing long-term complications associated with the condition [6,7]; however, the current treatment methods are limited to injury expansion prevention and rehabilitation only, lacking a mature and effective treatment for spinal cord injuries.

Stem cell therapy has been clinically implemented as a treatment for spinal cord injury, and the clinical results indicate promising therapeutic effects [8,9]. Particularly, mesenchymal stem cells (MSCs) have been extensively utilized in nerve repair due to their robust differentiation capacity and clinical safety [10,11]. Nevertheless, the application of MSCs still poses challenges, including concerns regarding cell viability and the uncertainty of cell differentiation. Consequently, developing an optimal microenvironment remains an effective approach for enhancing the effects of MSCs treatment in nerve repair. In recent years, researchers have initiated investigations into the potential utilization of MSCs in conjunction with diverse scaffolds to enhance their viability and cellular differentiation during transplantation for the treatment of SCI. In addition to their capacity for enhancing cell viability and differentiation, these scaffolds have demonstrated significant therapeutic effects, including the ability to fill diseased areas (providing physical support for axon regeneration), modulate immune responses, promote axon growth, and reduce scarring formation [12,13,14]. Previous research has demonstrated that the ordered arrangement of scaffold structure can efficiently direct the oriented growth of MSCs, significantly impacting their phenotype [15,16], proliferation [17], and differentiation [18,19,20]. However, it is noteworthy that most available scaffolds developed for loading MSCs fail to achieve satisfactory outcomes due to their inadequate physical and chemical properties, biological characteristics, cytocompatibility, or histocompatibility [21,22].

Recently, decellularized tissues or organs derived from porcine sources, such as small intestinal submucosa and heart valves, have been widely used in clinical practice. Therefore, the decellularized spinal cord (DSC) is considered to be a promising material for promoting tissue repair and nerve regeneration [22,23]. DSC is an extracellular matrix (ECM) derived from spinal cord tissue that effectively eliminates immunogenic components while retaining the extracellular matrix components found in natural spinal cord tissue. Porcine is a commonly used animal model, and obtaining its spinal cord is easy, which allows for the extraction of sufficient tissue for the preparation of a decellularized matrix for therapeutic use. However, after decellularization, the structure of the tissue will be destroyed. Recently, electrospinning techniques have demonstrated the ability to fabricate materials with an aligned structure, offering a promising approach for the preparation of materials that mimic the aligned architecture of spinal cord tissue [24,25]. And electrospinning has been used in preclinical studies to fabricate micro/nanofibrous scaffolds with aligned structures [26]. The aligned structures provide physical and topographic cues to MSCs, promoting the recruitment and migration of endogenous neural stem cells. Furthermore, the aligned structures guide the differentiation of MSCs towards neurons and promote the expression of neuron-related proteins [27]. However, fabricating porcine DSC into aligned fiber structures is rarely reported, and the effect of aligned and random DSC fiber on neuron regeneration after spinal cord injury has not been studied.

**Scheme 1:**
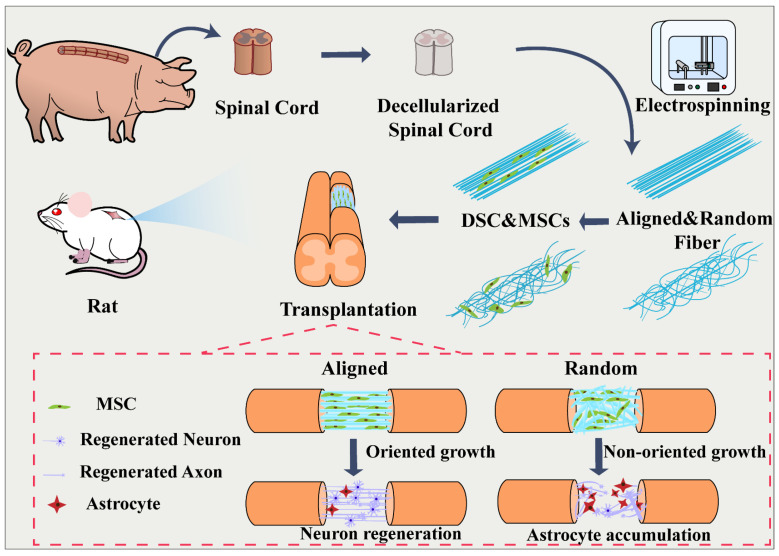
Preparation of aligned fibers and their application for SCI repair.

Herein, we developed a porcine DSC using a detergent-enzymatic method and subsequently fabricated DSC nanofibrous scaffolds through electrospinning (Scheme 1). After decellularization, the DSC effectively removed cellular immunogenic components while retaining the extracellular matrix proteins. Moreover, the nanofiber scaffolds exhibited an aligned structure and demonstrated the ability to enhance the adhesion, proliferation, and differentiation of MSCs by facilitating the alignment of MSCs on these nanofibers. The in vivo experiments demonstrated that the implantation of aligned nanofibers loaded with MSCs into SCI rats significantly enhances tissue regeneration and motor function recovery, compared to the random nanofibers group. In this study, we evaluated the therapeutic effect of aligned nanofibers on mesenchymal stem cell-based treatments for spinal cord injury, and the aligned nanofibers provide a promising strategy to fabricate aligned scaffolds for spinal cord injury treatment.

## 2. Materials and Methods

### 2.1. Preparation of DSC

We designed a novel decellularization process for porcine spinal cord tissues (SC) that combines physical, chemical, and biological methods. The fresh porcine spinal cord was rinsed with dH_2_O to eliminate blood and impurities. The intact spinal cord tissue was carefully selected and cut longitudinally into 8–10 mm segments while keeping the dura mater intact using a razor blade.

**Figure 1:**
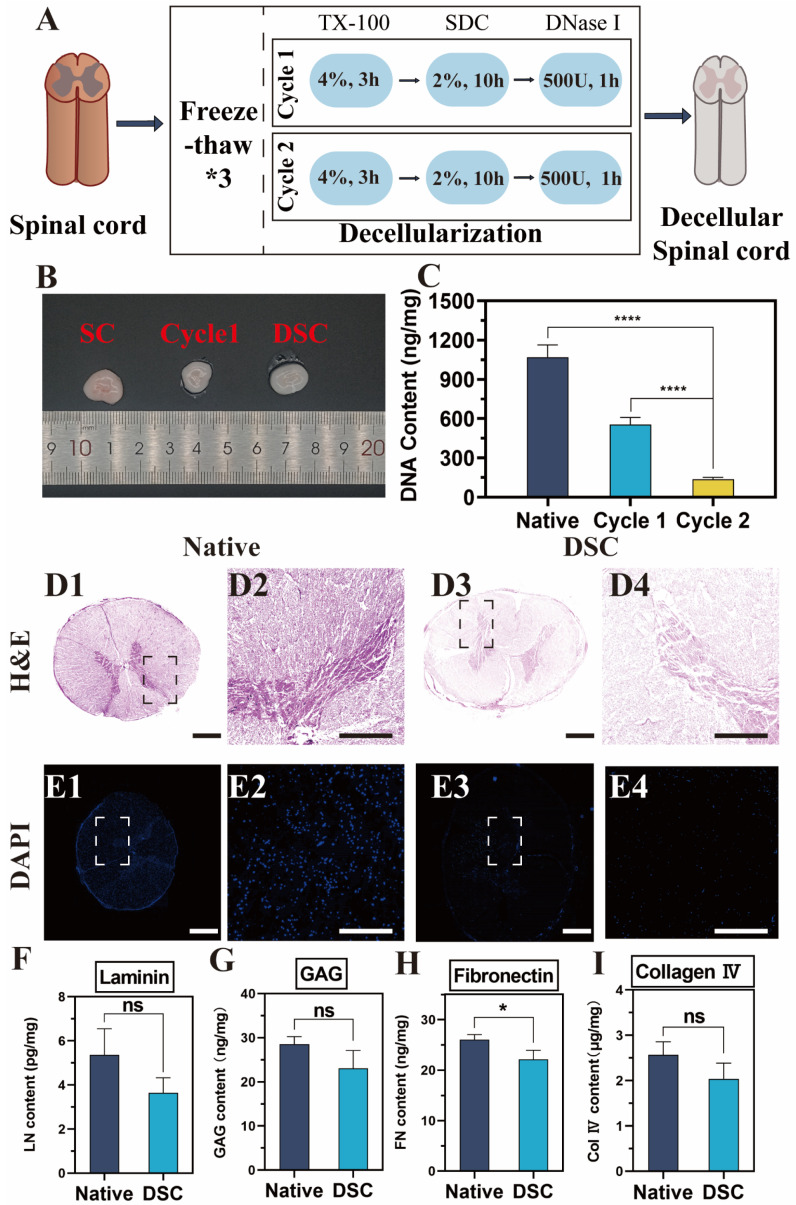
Fabrication and characterization of decellularized spinal cord tissue. (**A**) Schematic diagram of the decellularization process. (**B**) The visible morphology of decellularized spinal cord. (**C**) DNA content of spinal cord tissue (*n* = 3). (**D1**–**D4**) H&E staining (scale bar = 1 mm), (**E1**–**E4**) DAPI staining (scale bar = 1 mm), (**F**–**I**) LN, GAG, FN, COL IV protein content of decellularized spinal cord. The data are presented as Mean ± SD; * *p* < 0.05, **** *p* < 0.001; ns: no significant; data are representative of at least three independent experiments unless otherwise indicated.

Next, the tissues are subjected to a freeze–thaw cycle, which involves treating them at −80 °C for 1 h followed by 25 °C for 30 min, repeated three times. The tissues are washed with dH_2_O at 4 °C to remove impurities for 1 h. The cycle washes are conducted with a solution containing 4% TritonX-100 (Sigma-Aldrich, Saint Louis, MO, USA), 2% sodium deoxycholate (Sigma-Aldrich, Saint Louis, MO, USA), and 500 U/mL DNase (Sigma-Aldrich, Saint Louis, MO, USA), repeating twice. Each replacement of solvents necessitates rinsing with ultrapure water for 20 min, which is repeated three times (Figure 1A). All the solutions contained 2% Penicillin–streptomycin solution (Gibco, Grand Island, NY, USA), carried out in a thermostatic incubation shaker (THZ-103B, Yiheng, Shanghai, China) with set parameters of 70 rpm, 25 °C. Subsequently, the DSC is frozen in order to prevent tissue shrinkage.

### 2.2. Characterisation of DSC

To evaluate tissue decellularization and morphology, we fixed both SC and DSC in 4% PFA for 24 h. Afterward, we dehydrated, embedded, and sectioned the samples at a thickness of 5 μm. The sections were then stained using hematoxylin and eosin (H&E, ServiceBio, Wuhan, China) and 4′,6-diamidino-2-phenylindole (DAPI, ServiceBio, Wuhan, China) before being analyzed using fluorescence microscopy. To determine the quantity of DNA, we utilized DNeasy Blood & Tissue Kit (QIAGEN, Hilden, Germany) to extract DNA; each procedure was conducted according to the manufacturer’s instructions. The amount of extracted DNA was measured by spectrophotometer (NanoDrop 2000, Thermo Fisher Scientific, Waltham, MA, USA). Further, glycosaminoglycan (GAG), laminin (LN), collagen type IV (Col IV), and fibronectin (FN) proteins were quantified with enzyme-linked immunosorbent assays (Elisa, LanpaiBio, Shanghai, China).

### 2.3. Preparation of DSC/Gel Nanofiber Mat

The scaffolds were fabricated using electrospinning. Initially, we transformed the DSC into a powder through lyophilization and then prepared it for aqueous solution formation. The DSC solution comprised 6% DSC, 1.2% NaCI (Aladdin, Shanghai, China), and 0.1 wt% polyethylene oxide (PEO, Aladdin, Shanghai, China). To generate the spinning, we dissolved 6% Gelatin (Sigma-Aldrich, Saint Louis, MO, USA) in a hexafluoroisopropanol (HFIP, Aladdin, Shanghai, China) solution and blended the solutions in a 1:5 ratio. The spinning solution was maintained at 37 °C and mixed overnight through a magnetic stirrer to ensure the components were well combined.

Next, the electrospinning machine (SS-3556H, Ucalery, Beijing, China) utilized a spinning solution to fabricate the electrospun nanofiber mat. The parameters of the machine were set to an ambient temperature of 37 °C, humidity of 40%, propulsion speed of 1 mL/h, acceptance distance of 15 cm, and voltage ranging from 6 kV to 16 kV. Two specifications (300 rpm and 3000 rpm) were applied to create random and aligned fibers. Finally, fibers were placed in a temperature-drying oven (DHG-9023A, Dr. Storage, Shanghai, China) at 25 °C with 30% relative humidity for subsequent experiments.

Cross-linking of the electrospun nanofibers was conducted via glutaraldehyde (GA). Following the manufacture, the fibers underwent a 10 h treatment in a GA (Aladdin, Shanghai, China) atmosphere. Subsequently, the fibers were washed in anhydrous ethanol, 75% ethanol, and dH_2_O and then dried at room temperature.

### 2.4. Characterisation of DSC/Gel Nanofiber Mat

#### 2.4.1. Morphological Assessment

The Morphology of the fiber was evaluated via a scanning electron microscope (SEM, S-3400N, Hitachi, Tokyo, Japan) at an accelerating voltage of 10 kV; all samples were initially gold-coated (JFC-1600, JEOL, Tokyo, Japan) for 40 s and then investigated by SEM imaging. Twelve pictures were taken from various areas, each containing a minimum of 20 fibers. ImageJ (version 1.53) was employed to convert the photographs to 8-bit images, enabling the shape of each fiber to be outlined. DiameterJ and OrientationJ plugins were employed to measure the diameter and orientation of the fibers.

#### 2.4.2. Infrared Spectral Assessment

To conduct a structural analysis, the study utilized Fourier Transform infrared spectroscopy (FTIR, Nicolet 6700, Thermo Fisher Scientific, Waltham, MA, USA), operating within the 650–4000 cm^−1^ spectral range. This technique was applied to investigate uncrosslinked and cross-linked fiber mats, focusing on identifying and analyzing the positions of their respective absorption peaks.

#### 2.4.3. Thermogravimetric Assessment

To evaluate the impact of the cross-linking process on the stability features of fiber, a thermogravimetric analyzer (Q600, TA Instruments, New Castle, DE, USA) was utilized. The mass reduction of the films was determined by exposing them to a controlled thermal program, which escalated from 25 °C to 600 °C at a rate of 10 °C/min, all conducted under an atmosphere of nitrogen.

#### 2.4.4. Tensile Properties

The tensile properties of the samples were evaluated to investigate the effect of cross-linking on the tensile strength of the nanofibers. The tensile properties of the samples were measured by a universal testing machine (E44.204, MTS, Shanghai, China). The fiber mats were cut into standard samples with 5 × 40 mm dimensions rectangular strips. Each sample was mounted in a fixture, and its effective length was measured. We set the crosshead speed to 1 mm/h for the test. For each sample, five rectangular strips were prepared and tested, and the average values of ultimate tensile strength, tensile strain, and Young’s modulus were reported.

#### 2.4.5. Hydrophilicity and Wettability

The wettability and hydrophilicity of both uncross-linked and cross-linked electrospun nanofibers were assessed through the sessile drop method. Our procedure involved cutting the fiber membranes into 1 cm × 1 cm squares and taking three parallel samples from each group. Next, we added 10 μL of deionized water dropwise onto the surface of each sample. We captured photographs using a contact angle measuring instrument (JC2000D3, Powereach, Shanghai, China) to measure the contact angle size between the droplet and the scaffold surface.

#### 2.4.6. Degradation Properties

The degradability assessment of the fiber mat was evaluated by measuring the sample’s mass post-immersion. The initial mass of the lyophilized fibers was gauged before soaking in phosphate-buffered saline (PBS, ServiceBio, Wuhan, China) at 25 °C. The samples were removed at various intervals, lyophilized, and weighed. All degradation mediums are replaced weekly.

### 2.5. In Vitro Cell Experiments

#### 2.5.1. Cell Culture

MSCs (Cyagen, New Castle, DE, USA) were cultured in minimum essential medium α (MEM α, Gibco, Grand Island, NY, USA) containing 10% fetal bovine serum (FBS, Gibco, Grand Island, NY, USA) and 2% penicillin-streptomycin (P/S, Gibco, Grand Island, NY, USA) in a humidified incubator at 37 °C and 5% CO_2_.

To prepare the nanofiber for cell seeding, we first cut the fiber mats into 5 × 5 mm^2^ squares and sterilized them with UV light for 10 h while pressing a metal ring against them. Once the fibrous membranes were thoroughly sterilized, we added 20,000 MSCs to each well of a 24-well polystyrene cell culture plate, waited 20 min for the cells to attach to the fibers, and then supplemented with 500 μL of cell culture medium.

#### 2.5.2. Cell Cytocompatibility Assessment

The viability and proliferation of cultured cells on fiber mats were assessed using Cell Counting Kit-8 (CCK-8, ServiceBio, Wuhan, China). Prior to cell culture, the mats were prepared into uniform sizes in a centrifuge tube, treated with 75% alcohol for 10 min, and sterilized with UV for 1 h. To obtain the extract, the centrifuge tube containing the mats was added to Dulbecco’s modified eagle medium (DMEM, Gibco, Grand Island, NY, USA) (10% FBS + 100 U/mL P/S) at a ratio of 1 cm^2^/mL and placed in a 37 °C, 5% CO_2_ incubator for 24 h. The extract was then passed through a 0.22 μm microporous filter membrane to remove bacteria. MSCs were cultured using DMEM (10% FBS + 100 U/mL P/S), extract, and DEME (10% DMSO), respectively, following the culture method described in Section 2.5.1.

CCK-8 solution was added to the cells cultured on the medium at three different time points: 1 day, 3 days, and 7 days. After incubation for 1 h, the absorbance at 450 nm was measured using a Microplate Reader (SPECTR Amax 384, Molecular Devices, San Jose, CA, USA). The mean absorbance readings were used to calculate the cell count through the calibration curve. The total number of cells at each time point was calculated from three independent triplicate experiments for all samples.

MSCs were seeded into 96-well plates at a density of 5 × 10^2^ cells per well. The plates were then incubated for one day The experimental group received DSC/Gel fiber membrane extract, while the control group was maintained in a DMEM medium. The plates were incubated for one, three, and seven days, respectively. At the corresponding time points, cells were stained by adding staining solution, incubating for 30 min, adding PBS, and washing twice. Laser confocal microscopy was subsequently performed. Cells were incubated for 30 min, washed twice, observed under a confocal laser microscope, and photographed.

#### 2.5.3. Cell Growth Direction Assessment

Initially, 1 × 10^6^ cells were seeded onto each ordered and unordered DSC/Gel membrane within a 24-well plate, following the specifications outlined in Section 2.5.1. The plate was then placed in a cell culture incubator maintained at 37 °C to ensure optimal growth conditions. On the subsequent day, the cells were stained with 200 μL of FITC Phalloidin working solution. The stained cells were then observed and photographed using a laser confocal microscope (TCS SP8, Leica, Wetzlar, Germany).

To assess the effect of fiber structure on the migration of MSCs, we conducted cell-scratching experiments. Initially, 5 × 10^5^ cells were seeded into each well of a 6-well plate, and the medium was replaced after 6 h. The following day, the cells were scratched with a pipette tip perpendicular to the horizontal line behind them. The surface of the scratch was then coated with either an ordered or an irregular fiber membrane. In the ordered group, the direction of fiber orientation was designed to be perpendicular to the direction of the scratch. Serum-free medium was added, and the plates were incubated. At 0 h, 12 h, 24 h, and 36 h, images were captured under a microscope to document the results.

#### 2.5.4. In Vitro Differentiation of MSCs

To detect gene expression of MSCs neural differentiation stimulated by nanofiber, we conducted a quantitative real-time polymerase chain reaction (qRT-PCR) assay. For each group on days 1, 3, and 7, we isolated and extracted the total RNA using TRIzol reagent (TaKaRa, Kyoto, Japan). Then, we prepared first-strand cDNA by reverse transcription using a Primerscript RT kit (TaKaRa, Kyoto, Japan) and a gene amplifier (Tgradient, Biometra, Göttingen, Germany). The first-strand cDNA was then amplified in a real-time fluorescence quantitative PCR instrument (CFX96Touch, Bio-Rad, Hercules, CA, USA) using TBGreen (TaKaRa, Kyoto, Japan) for qRT-PCR. We quantified glyceraldehyde-3-phosphate dehydrogenase (GAPDH) and six genes of interest (see Table 1) with a hot start denaturation step at 95 °C for 30 s and then recorded fluorescence intensity during 40 cycles at 95 °C for 5 s and 60 °C for 30 s. We normalized the relative transcript expression levels of the target gene to GAPDH and expressed them as the means ± SD (*n* = 3 for each group). Finally, we analyzed the data using the 2^−ΔΔCt^ method.

### 2.6. In Vivo Animal Experiments

The experiments used 30 female Sprague-Dawley (SD) rats weighing 180–200 g and of SPF grade. All animal experimental protocols were approved by the Experimental Animal Ethics Committee of East China University of Science and Technology (ECUST-2020-07001) and met the requirements of the National Institutes of Health Guide for the Care and Use of Laboratory Animals.

#### 2.6.1. Preparation of MSCs@DSC/Gel

The method described in Section 2.5.1 was employed to load MSCs onto the fiber membrane. Following a three-day incubation, the fiber mat was removed and cut into 2 mm lengths, yielding MSCs@DSC/Gel.

#### 2.6.2. Animal Surgery

In the standard SCI model, female SD rats underwent a T10 lateral hemisection procedure. The procedure involved administering pentobarbital sodium (50 mg/kg) via intraperitoneal injection and isoflurane via inhalation (RWD, Shenzhen, China) to anesthetize the rat. A 2 mm incision was made on the right side of the spinal cord at the T10 segment using a razor blade, causing injury. Aligned or random MSCs@DSC/Gel were implanted into the center of the injured area, while the SCI group was left untreated. The incision was then sutured, and the rat was allowed to recover from the injury. Postoperative penicillin therapy was administered once a day for two weeks, and artificial urination was provided twice daily until the rat regained urinary function.

#### 2.6.3. Histological Analysis

To analyze the neurogenesis effect of the MSCs@DSC/Gel, rats that have undergone surgery were sacrificed after 8 weeks, and their spinal cords were extracted, fixed, and embedded. The samples were then sliced into 5 μm thick sections along the implant’s longitudinal axis and stained with H&E (ServiceBio, Wuhan, China) to observe tissue formation across the different groups. Immunohistochemical staining was conducted on the sections using a range of antibodies, including anti-Map-2 (production in rabbit, 1:100, Sigma-Aldrich, Saint Louis, MO, USA), anti-GFAP (production in mouse, 1:100, Sigma-Aldrich, Saint Louis, MO, USA), goat anti-rabbit IgG (Millipore, Billerica, MA, USA), and goat anti-mouse IgG (Millipore, Billerica, MA, USA). Primary antibodies were incubated overnight at 4 °C, then washed with PBS before secondary antibodies were added and incubated at 37 °C for 30 min. Streptavidin-HRP was added for 20 min, followed by DAPI (ServiceBio, Wuhan, China) to visualize antibody binding sites. The stained sections were imaged using a laser confocal microscope (TCS SP8, Leica, Wetzlar, Germany), and fluorescence intensity was analyzed using ImageJ software.

#### 2.6.4. Functional Behavior Evaluation

The motor function was evaluated weekly by open-field testing using the Basso–Beattie–Bresnahan (BBB) score. Three unbiased observers assessed hindlimb motor recovery on a scale of 0 to 21, where 0 represents no hind-limb movement, and 21 signifies normal motor function. The BBB score was documented from the injury until 8 weeks later.

### 2.7. Statistical Analyses

GraphPad Prism 9.0 (GraphPad, San Diego, CA, USA) and Adobe Illustrator 2021 (Adobe, San Jose, CA, USA) were used to generate graphs. ImageJ (Wayne Rasband, National Institutes of Health, Bethesda, MD, USA) was used to analyze SEM, IF, and H&E images quantitatively. Numbers indicate biological replicates of experiments performed at least three times unless otherwise indicated. All data were represented as mean ± SD. Error bars indicated SD (* *p* < 0.05, ** *p* < 0.01, *** *p* < 0.005, **** *p* < 0.001). The significance level was set at *p* ≥ 0.05.

## 3. Results

### 3.1. Fabrication and Characterization of DSC

To create a regenerative microenvironment at the SCI site, we developed a decellularized matrix from porcine spinal cord tissue. The process, illustrated in Figure 1A, includes the freeze–thaw method, detergent process and bio-enzyme process to remove cellular components. After decellularization, the spinal cord tissue experienced significant histomorphological changes, as evidenced by the hyalinization depicted in Figure 1B. Removing residual cell nuclei is crucial when transplanting allogeneic material to avoid triggering immune responses and causing tissue damage [28,29]. To determine the effectiveness of our decellularization process in removing DNA content, the qualitative analysis using H&E and DAPI and the quantitative characterization of DNA content were performed. According to the findings presented in Figure 1C, a noticeable reduction in DNA content was observed, decreasing from 1069.84 ± 37.88 ng/mg in the Native group to 554.35 ± 33.35 ng/mg (Cycle 1) and 137.34 ± 12.66 ng/mg (Cycle 2). The DNA removal rate of 87% indicates the effectiveness of the combined chemical (SDC and TX-100) and enzymatic (DNase I) treatments in removing DNA from spinal cord tissues.

**Figure A1:**
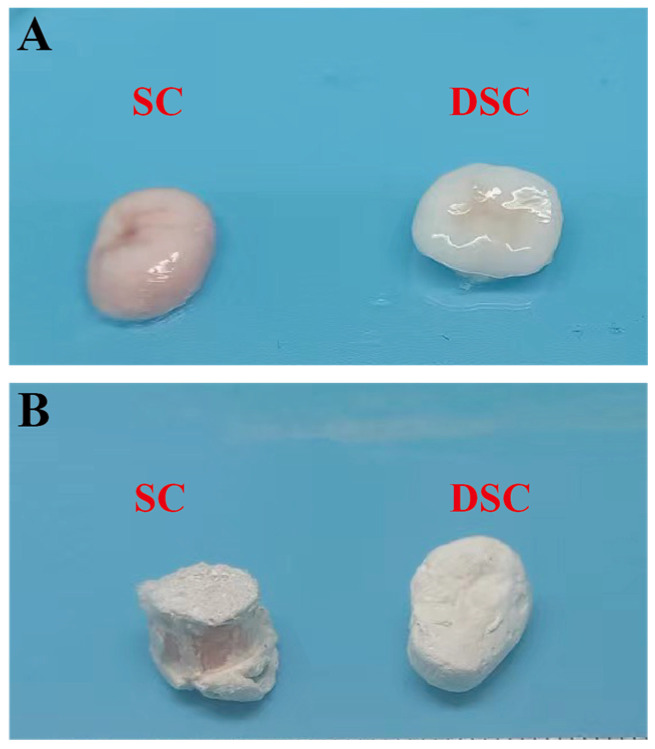
Morphology of SC and DSC (**A**) before and (**B**) after lyophilization.

Moreover, we performed H&E and DAPI staining on cells in both SC and DSC, respectively. Figure 1D,E display a gradual reduction in blue dots in the tissues, indicating successful cell removal. Additionally, the quantitative analysis of proteins linked to nerve regeneration was further performed. Figure 1F–I illustrate the retention average percentages of various proteins following the decellularization process. Specifically, the quantities retained for LN, GAG, FN, and Col IV were documented at 67.91%, 81.02%, 84.95%, and 79.38%, respectively. Except for the FN group, which exhibited a significant decrease, the protein content across the other groups demonstrated no significant differences. Therefore, we have concluded that the decellularization technique effectively eliminated the immunogenic components while more comprehensively preserving the essential elements of the ECM in spinal cord tissues. All quantitative characterizations are based on the dry weight state of the spinal cord (Figure A1). This discovery provides a foundation for subsequent applications of the DSC in SCI repair.

### 3.2. Fabrication and Characterization of DSC/Gel Nanofiber Mat

**Figure 2:**
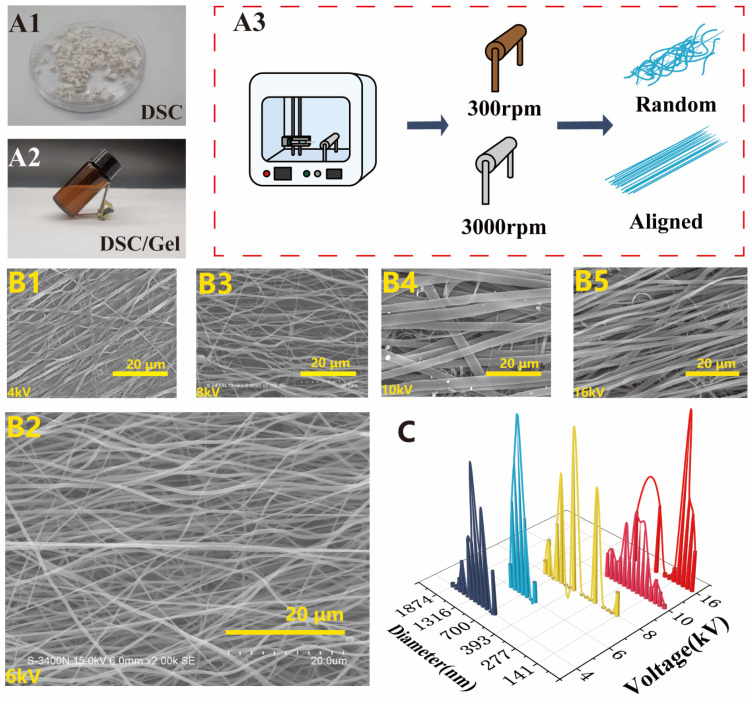
Preparation of DSC/Gel nanofiber mat. (**A**) Configuration of the solution (**A1**,**A2**) and the preparation of aligned and random fibers (**A3**). (**B1**–**B5**) SEM images of fibers under 4–16 kV process. (scale bar = 20 μm) (**C**) Fiber diameter distribution statistics. The data are presented as Mean ± SD.

**Figure A2:**
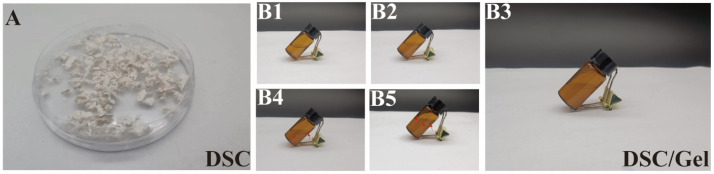
(**A**) DSC and (**B**) solutions of different concentrations ((**B1**): 0%, (**B2**): 0.1%, (**B3**): 1%, (**B4**): 5%, (**B5**): 10%).

**Figure A3:**
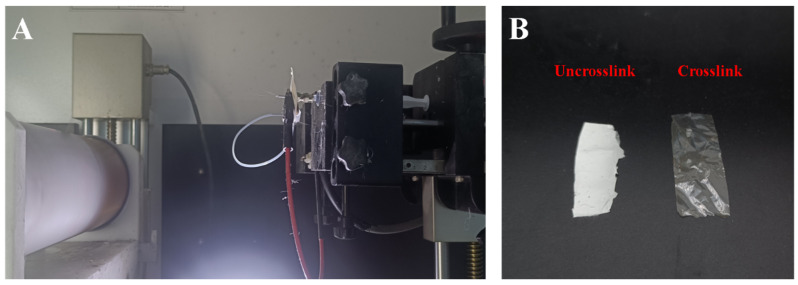
(**A**) Electrospinning equipment and (**B**) material morphology before and after cross-linking.

Given the challenges of DSC in processing through electrostatic spinning, our approach utilized biocompatible gelatin to fabricate composite fibers incorporating DSC. As illustrated in Figure A2, dissolution experiments revealed that the dispersion of DSC in HFIP is enhanced when the concentration is 1%. Furthermore, an aqueous phase was introduced strategically into the organic solvent to improve the dispersion characteristics of the DSC solution. The spinning solution was formulated by mixing DSC powder with gelatin, intended for subsequent processes, and the details of the spinning solution are illustrated in Figure 2A. Our research utilized orthogonal experiments focused on two key factors: the DSC/Gel ratio and the HFIP/H_2_O ratio. Then, we assess the consistency of the solution and the stability of the spinning process. As shown in Table 2, mixing DSC with gelatin can enhance the processability of the spinning solution and the ability to dissolve to form fiber. Moreover, increasing the water ratio in the solvent can also raise solubility and fluidity, but it may inhibit fiber formation during the spinning process due to unfavorable solvent evaporation. We found that a DSC/Gel ratio of 1:3 and an HFIP/H_2_O ratio of 5:1 resulted in a uniformly dispersed spinning precursor solution with maximum DSC concentration (Figure A2). Subsequent work was based on the solution. The electrostatic spinning process was conducted within a chamber (Figure A3), with the spinning process being controlled at an ambient temperature of 37 °C, relative humidity of 40%, and a drive rate of 1 mL/h. Previous studies have suggested that scaffolds with fiber diameters of around 750 nm are most effective for axon-oriented extension [30,31]. SEM images of the fibers are shown in Figure 2(B1–B5); the spinning voltage was varied to achieve different diameters of the fiber membranes. The statistical results of the fiber diameters are presented in Figure 2C, with a diameter of 1.08 ± 0.33 μm (4 kV), 0.72 ± 0.30 μm (6 kV), 0.41 ± 0.26 μm (8 kV), 0.31 ± 0.20 μm (10 kV) and 0.14 ± 0.10 μm (16 kV), the fiber diameter gradually decreases as the voltage increases. The fiber diameter uniformity is satisfactory, and the subsequent process is determined at about 6 kV.

**Figure 3:**
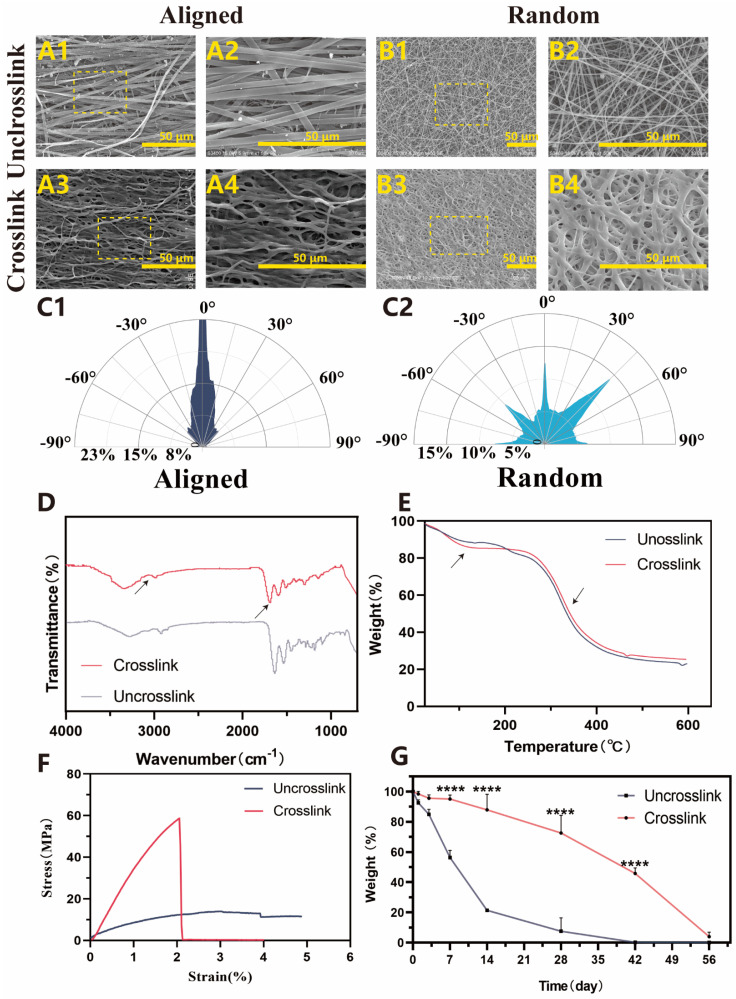
Preparation of DSC/Gel nanofiber mat. (**A1**–**A4**) SEM images of aligned fibers before and after cross-linking (scale bar = 50 μm). (**B1**–**B4**) SEM images of random fibers before and after cross-linking (scale bar = 50 μm). (**C1**,**C2**) Fiber orientation statistics of the aligned group and random group. (**D**) FTIR and (**E**) TGA curve of fiber (**F**) Tensile properties of fibers. (**G**) Fiber degradability. The data are presented as Mean ± SD; **** *p* < 0.001; ns: no significant; data are representative of at least three independent experiments unless otherwise indicated.

DSC/Gel is weak to water exposure and lacks the necessary strength for stem cell loading and implantation in vivo as a tissue engineering scaffold; we opted to enhance its mechanical durability and stability by introducing GA (Figure 3B) into the nanofiber mats [32]. To verify the impact of GA on the molecular bonding of the fibers, we conducted Fourier transform infrared spectroscopy (FTIR) and thermogravimetric analysis (TGA). The GA process involves a reaction between the amino group in gelatin and the aldehyde group in GA. As illustrated in Figure 3D, the amide A band at approximately 3300 cm^−1^ can be attributed to gelatin’s vibrational absorption of –NH and –OH. The amide I band at around 1660 cm^−1^ indicates the characteristic spectral band associated with the stretching vibration of C=O and Schiff base (C=N). The amide II band, situated at approximately 1540 cm^−1^, is attributed to the stretching vibration of C–N and the bending vibration of N–H in protein. The amide III band, located at approximately 1230 cm^−1^, is associated with the stretching vibration of C–N and the bending vibration of N–H in gelatin. After treatment by glutaraldehyde, a blueshift for the amide A band and amide II band is observed. This suggests that glutaraldehyde may interact with the amino group in gelatin, potentially leading to alterations in the secondary structure of gelatin proteins and subsequent improvements in the material properties. The TGA results (Figure 3E) revealed that the Cross-link group lost weight more in the 25 to 240 °C range. The inability of gelatin molecules to form hydrogen bonds with the water molecules following the GA treatment may be responsible for this observation, leading to a decreased ability to bind with water molecules. Moreover, the final weight rate of the fiber membrane increased from 23% to 25% after cross-link, indicating an improvement in thermal stability. The FTIR and TGA results demonstrated that the chemical bonding of fibers transformed due to the GA treatment.

Previous investigations have elucidated that the orientation of nanofibers along a single axis can influence the directional growth of cells and foster the differentiation of stem cells into neuronal phenotypes [33]. In our study, two different receivers, one spinning at 300 rpm and the other at 3000 rpm, were utilized to produce random and aligned fibers, respectively. SEM analysis (Figure 3A,B) revealed that the diameters of both fiber groups were concentrated between 700 nm and 900 nm, with no notable difference in diameter between the two. However, the fibers collected by the 300 rpm receiver were more haphazardly arranged, in contrast to the aligned structure of those collected by the 3000 rpm receiver. This indicates that using a high-speed collector can significantly enhance material orientation. Additionally, comparing cross-linked and un-crosslinked fibers demonstrated that GA treatment can create a mesh structure between the fibers, and the aligned fibers preserved their orientation after cross-linking. To evaluate the fiber orientation in a precise manner, we conducted angle measurements of the fibers in SEM images. As depicted in Figure 3C, our findings indicate that the proportion of fibers in the Aligned and Random groups, within the angle range of −30° to 30°, is 76.27% and 32.62%, respectively. This observation suggests that the distribution of fibers in the 3000 rpm group is highly concentrated, with an apparent inclination towards orientation.

Tensile property analyses in Figure 3F illustrate the mechanical characteristics of the uncross-linked and cross-linked groups. The application of GA has significantly elevated the tensile strength of the fiber mat from 12 MPa to 58 MPa, achieving a modulus comparable to that of the natural spinal dura mater, which is 48 MPa [34]. To test the degradability, we immersed the mat in PBS for 8 weeks at 37 °C to assess its degradation properties. As shown in Figure 3G, during the initial three-day period of the experiment, no notable discrepancy was observed in the degradation performance of the two groups (Uncrosslink: 84%, Crosslink: 95%). On the seventh day, the Uncrosslink group exhibited a 50% reduction in weight, whereas the Cross-link group demonstrated a 10% reduction. However, crosslinking improves the stability of the material, with a considerable amount (72%) of the structure remaining intact after 4 weeks (Uncrosslink: 7%)—furthermore, the degradation of the material until the eighth week of the experiment. The alteration in the degradation rate is predominantly influenced by the modification in the structure of the macromolecules. Subsequent to GA treatment, a robust cross-linked network structure is established between the molecules, thereby extending the degradation period of the fiber membrane. The results demonstrated that the mechanical properties and stability of the fibers were enhanced by GA treatment, thus establishing a foundation for subsequent applications.

Consequently, a ratio of the spinning solution has been identified through experimentation. We have successfully fabricated an aligned fiber mat by adjusting the voltage, modifying the collector, and cross-linking. The mat exhibits suitable mechanical properties and stability, supporting the subsequent loading of MSCs.

### 3.3. DSC/Gel Promotes MSCs Directional Growth and Differentiation

**Figure 4:**
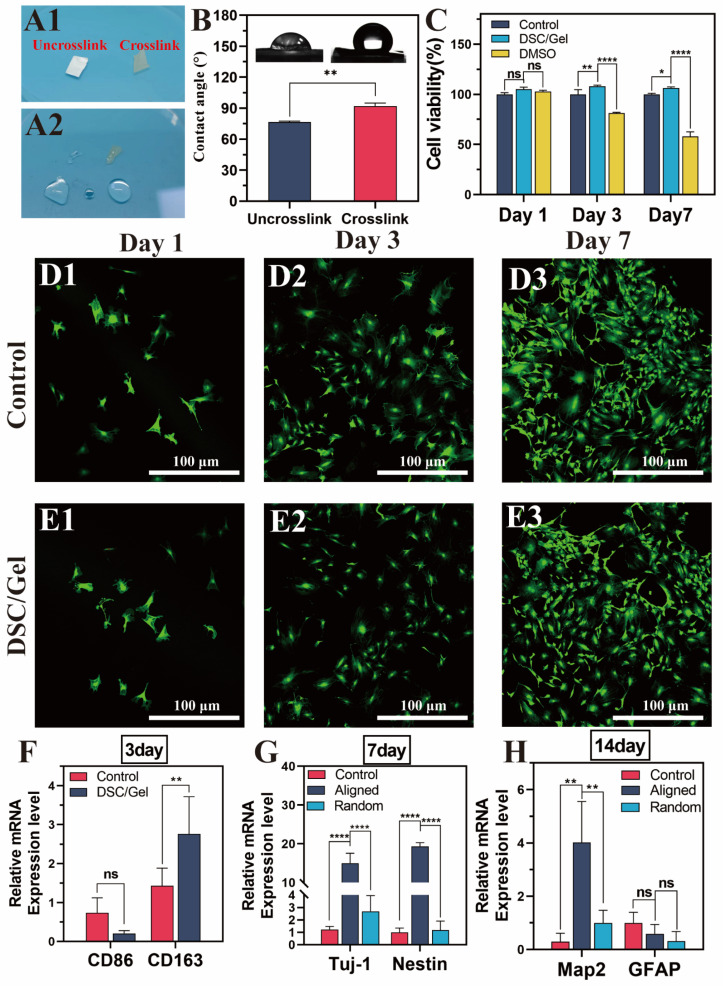
In vitro cellular experiments. (**A1**,**A2**) Pictures of cross-linked and uncross-linked groups before (**A1**) and after (**A2**) contact with water. (**B**) Fiber mat contact angle test. (**C**) CCK-8 and (**D1**–**D3**,**E1**–**E3**) cell live-dead staining of MSCs at day 1, day 3, and day 7. (**F**–**H**) Comparative analysis of mRNA expression levels of markers, CD86, CD163, Tuj-1, Nestin, Map-2, and GFAP in MSCs at day 3 (**F**), day 7 (**G**), and day 14 (**H**) The data are presented as Mean ± SD; * *p* < 0.05, ** *p* < 0.01, **** *p* < 0.001; ns: no significant; data are representative of at least three independent experiments unless otherwise indicated.

The intrinsic elevated solubility of the DSC fiber mat weakens its utility in supporting MSCs co-culture [35]. Nevertheless, we have devised a cross-linking technology method to overcome this limitation. The macroscopic state of the uncross-link and cross-link group before and after contact with water is shown in Figure 4A. The results reveal that the uncross-linked fiber mat shrinks in volume and loses structure. In contrast, the cross-linked fiber maintains its structure and fiber morphology, significantly improving the stability of the material. Contact angle data in Figure 4B supports the conclusion. The contact angle is from 76.47° ± 0.64° to 91.99° ± 3.00° by GA treatment, which increases its hydrophobicity and improves its stability. The improvements ensure that the mat can better adapt to the needs of loading MSCs.

The survival of MSCs was evaluated using the CCK-8 reagent, and the results are presented in Figure 4C. On day 1, there was no significant difference in cell activity between the Control group (100.00% ± 1.34%), the DSC/Gel group (105.07% ± 2.29%), and the DMSO group (102.85% ± 1.34%). Over time, on days 3 and 7, the cellular activity of the DSC/Gel group demonstrated a significant advantage over that of the control group. On day 3, the cellular activity of the DSC/Gel group was 108.12% ± 1.25%, while on day 7, it was 106.35% ± 1.06%. The experimental group demonstrated a significant advantage over the control group, whereas the positive control group exhibited significant cytotoxicity (3 days: 81.09% ± 0.99%, 7 days: 58.03% ± 4.53%). These findings indicate that the DSC/Gel fiber membrane has the capacity to stimulate the proliferation of MSCs, thereby demonstrating favorable biocompatibility. The results of live-dead cell staining (Figure 4D,E) indicated that the number of live MSCs in the field of view was comparable to that of the control group (normal medium) and that the cell morphology did not change significantly. Moreover, no significant cell death was observed in either the control or DSC/Gel groups. This indicated that the DSC/Gel fiber membrane exhibited favorable biocompatibility, which was consistent with the results of the CCK-8 assay.

To explore the influence of fiber on MSCs differentiation, we implant MSCs on fiber scaffolds. qRT-PCR was utilized to evaluate the gene expression levels associated with CD86, CD163, Tuj-1, Nestin, Map-2, and GFAP following the induction of MSCs. Figure 4F–H showed no significant difference in the pro-inflammatory factor CD86 after 1 day of culture. However, the anti-inflammatory factor CD163 was up-regulated (93%), indicating that the nanofiber mat did not cause significant cellular inflammation. After 3 and 7 days, neurotrophic and neural differentiation genes were up-regulated in the Aligned DSC/Gel-induced MSCs (Tuj-1:1121%, Nestin:1832%, Map-2:1240%), demonstrating the ability to induce MSCs to neural cell differentiation.

**Figure 5:**
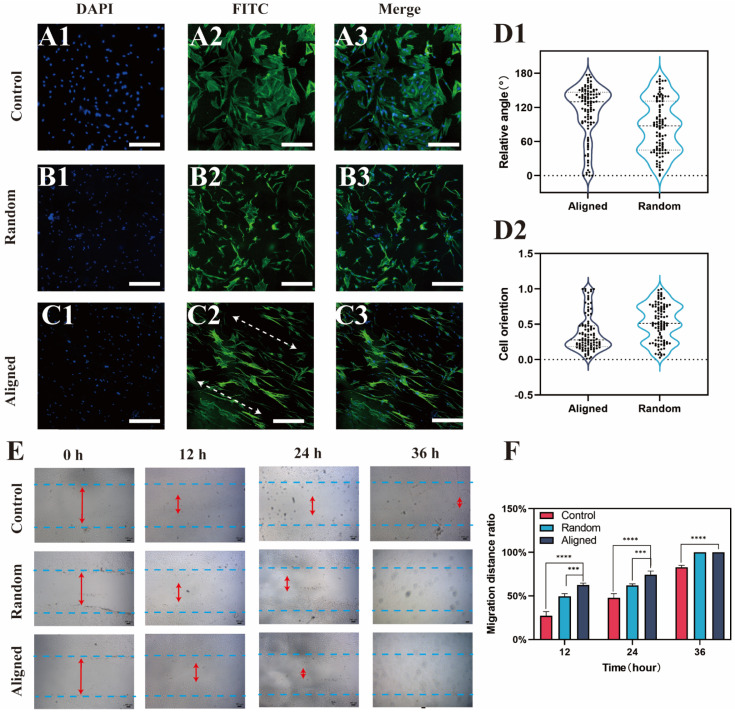
Cell growth assessment. (**A1**–**A3**,**B1**–**B3**,**C1**–**C3**) Cytoskeleton and DAPI images of MSCs after 1 day of incubation on fiber mat (scale bar = 200 μm). (**D1**,**D2**) Distribution of offset angle and orientation of MSCs in aligned and random groups. (**E**,**F**) Images and distance statistics of MSCs migrating (scale bar = 100 μm). The data are presented as Mean ± SD; *** *p* < 0.005, **** *p* < 0.001; ns: no significant; data are representative of at least three independent experiments unless otherwise indicated.

To assess the role of fiber membrane structure on MSCs growth direction, we inoculated MSCs onto fiber membranes and cultured them for one day. Subsequently, we stained the MSCs cytoskeleton using FITC Phalloidin and observed it with confocal microscopy. The results demonstrated that the MSCs in the Aligned group exhibited a distinct convergent growth pattern (Figure 5A–C), suggesting that the nanofiber structure of the aligned fibrous mat profoundly influences cell spreading morphology and growth direction. Additionally, the close contact between the cells in this group may facilitate intercellular information exchange. Further observation of enlarged images revealed that cells in the random fiber membrane exhibited a multipolar morphology, while cells in the aligned membrane extended in the direction of the fibers (white arrows). This suggests that fiber topology may guide cell extension. To provide a more intuitive characterization of cell orientation, we counted the offset angle of the cells and calculated the orientation α. Figure 5D illustrates the offset angle and orientation of the cells in the Aligned and Random groups, respectively. It was observed that the orientation of the cells in the Aligned group was more concentrated, with 62% of the cells having an offset angle of 100°–160° (α = 0.44–0.11), while the cells in the Random group had a more dispersed offset angle. This indicated that the aligned fiber structure could better guide MSCs to align and grow directionally.

A comparison was conducted to assess the impact of aligned DSC/Gel fiber membranes (Aligned) and random DSC/Gel fiber membranes (Random) on the migratory capacity of MSCs. The results demonstrated that the width of the scratches (red arrows) in the Aligned group significantly reduced with increased incubation time beyond 36 h, compared to the Control and Random groups (Figure 5E,F). This indicates that the fiber topology of the ordered DSC/Gel fiber membrane facilitated MSCs migration. At 24 h post-scratch, the proportion of cell migration distance was 47.80% ± 4.96% in the Control group, 62.11% ± 1.56% in the Random group, and 74.43% ± 4.41% in the Aligned group. At 36 h post-scratch, the Aligned and Random groups had formed a dense layer at the scratch location, unlike the Control group. The Aligned group exhibited a superior healing phenomenon compared to the other groups, suggesting that the introduction of an ordered nanofiber structure enhanced the migratory behavior of MSCs along the fiber direction.

Overall, we concluded that the DSC/Gel is cytocompatible and that the aligned fiber structure can guide the MSCs to grow directionally and differentiate into neurons, creating a favorable environment for MSCs and offering a promising treatment for SCI.

### 3.4. MSCs@DSC/Gel Facilitates Tissue Regeneration and Motor Function Recovery

**Figure 6:**
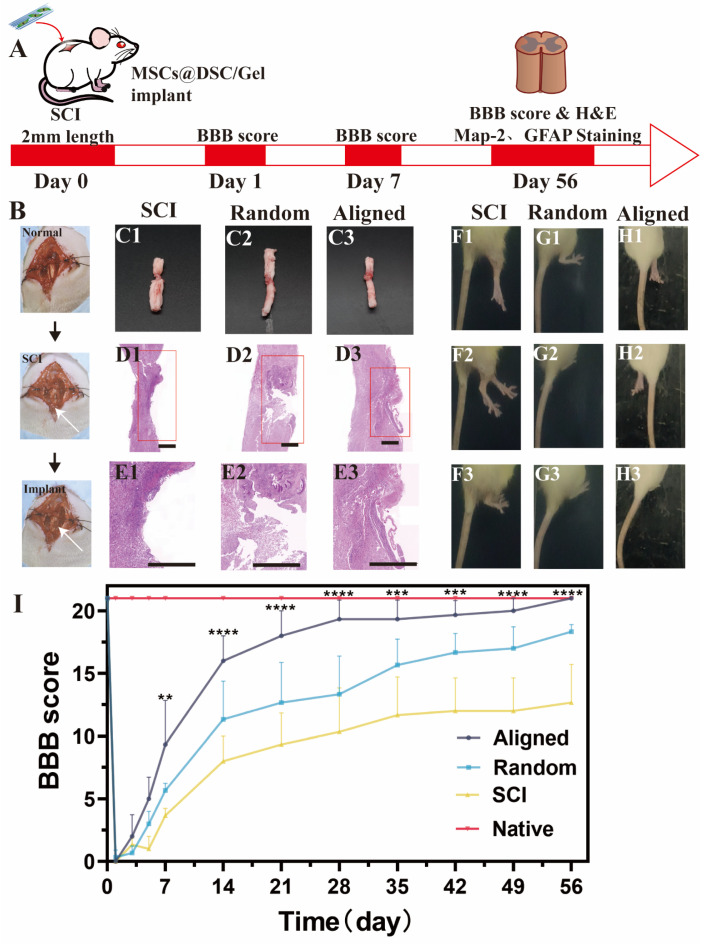
Fiber implantation in vivo experiments. (**A**,**B**) The timeline of SCI modeling and histological characterization. (**C1**–**C3**) Spinal cord appearance and morphology. (**D1**–**D3**,**E1**–**E3**) H&E staining of the spinal cord in SCI, Random, and Aligned groups (scale bar = 1 mm). (**F1**–**F3**,**G1**–**G3**,**H1**–**H3**) Behavioral assessment of rats after surgery. (**I**) The BBB score of the right hind limb in three groups after right lateral spinal cord hemisection. The data are presented as Mean ± SD; ** *p* < 0.01, *** *p* < 0.005, **** *p* < 0.001; ns: no significant; data are representative of at least three independent experiments unless otherwise indicated.

To investigate the effect of aligned fiber on SCI repair, we established a 2 mm hemisection model of the right side of the T10 segment of the spinal cord in SD rats. MSCs-encapsulated (2 × 10^4^ cells) Aligned DSC/Gel mat (Aligned) was implanted into the lesion site as an experiment group. The groups that used Random MSCs@DSC/Gel mat (Random) and Unoperated (SCI) were used as controls. In order to assess the efficacy following aligned fiber implantation, we employed the use of photography and recording to document the movement of the hind limbs of rats (Figure 6B); it revealed that the SCI group had poor mobility in the hip and knee joints, the Random group had movable joints but no continuous support for the paws, and the Aligned group had more coordinated movements compared other groups. The appearance of the spinal cord and H&E staining after surgery is depicted in Figure 6C–E. To assess the impact of tissue repair, tissues were extracted after 8 weeks and subjected to H&E staining. The histological staining indicated that the SCI group had sparse neural tissues at the SCI, with the largest cystic cavity area among the three groups. The Random group had a reduced area of the cystic cavity, and the neural tissue structure at the injury site was heterogeneous. The Aligned group showed a noticeable repair effect on the damaged area, with new neural tissue connection and integration making the new nerve tissue more efficient. In the Aligned group, tissue was effectively repaired at the injury site, with new nerve tissues being interconnected and integrated with the original tissues on both sides of the injury.

In the present study, the Basso–Beattie–Bresnahan (BBB) score is employed as a primary metric for evaluating motor recovery after SCI in rats [36,37]. In our research, the “Native” group represents sham-operated rats, the “SCI” group represents rats with only hemisection injury modeling, the “Random” group represents those with MSCs@random DSC/Gel fibrous membrane implantation, and the “Aligned” group represents those with MSCs@aligned DSC/Gel fibrous membrane implantation. Figure 6F–H presents a schematic diagram of the locomotor status of the rats’ hind limbs in different groups at 8 weeks post-surgery. The SCI group exhibited poorer hind-limb joint mobility and was capable of unbalanced weight-bearing forward locomotion. The Random group demonstrated improved locomotor coordination, although the hind feet could not remain parallel to the direction of movement. In contrast, the Aligned group exhibited the best locomotor status, characterized by a smooth gait and both paws remaining parallel to the direction of movement when raised. Figure 6I presents the detailed BBB score data. The rats’ locomotion progressively improved over time, with the Aligned group showing the most favorable recovery, followed by the Random group, and the SCI group showing the least favorable results. During the initial 7 days, there was no discernible difference in the scores among the three groups. However, between the 7th and 14th days, the BBB scores of the three groups showed notable improvement, with the Aligned group demonstrating a 7-point increase, the Random group a six-point increase, and the SCI group a five-point increase. The motor functions of the rats in the Aligned group were restored, showing significant differences among the groups. On the 28th day, the BBB scores of the rats in the Aligned group had reached 19 points, and the BBB scores of the SCI group had reached 13 points. By the 28th day, the BBB score of rats in the Aligned group had reached 19, indicating significant improvement in motor function compared to the Random and SCI groups, which had BBB scores of 13 and 10, respectively. These findings suggest that the implantation of aligned mats may facilitate spinal cord regeneration at the injury site and promote motor function recovery.

### 3.5. MSCs@DSC/Gel Promote Neuronal Regeneration and Inhibit Glial Scarring

**Figure 7:**
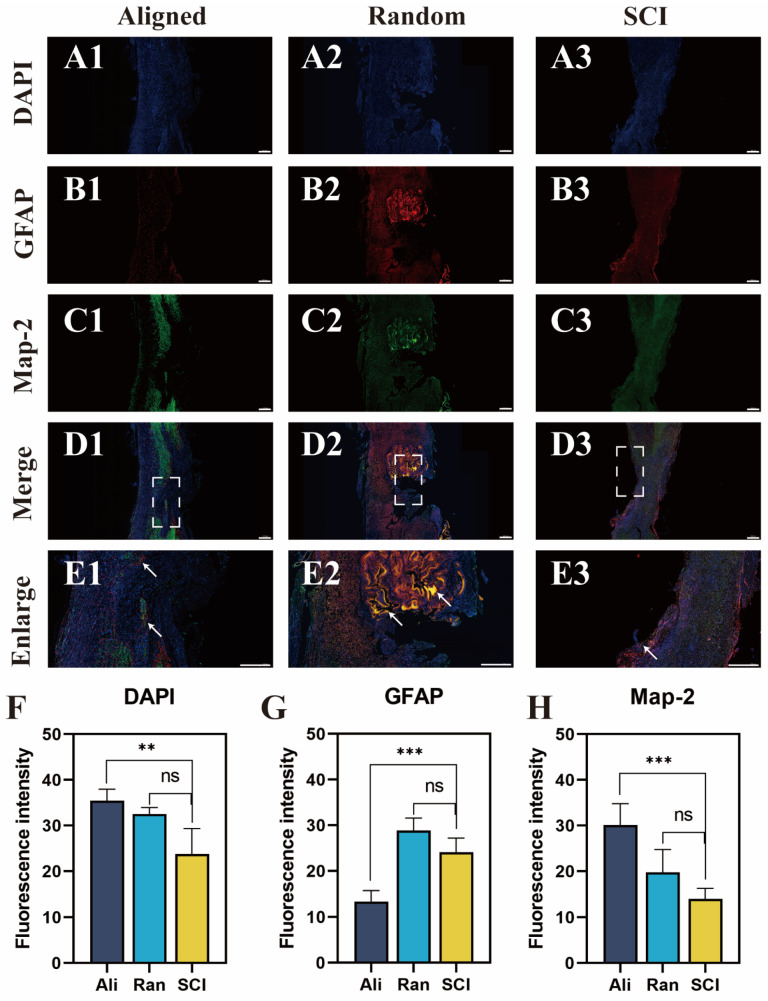
Aligned scaffolds promote neuronal regeneration and inhibit glial scarring. (**A1**–**A3**,**B1**–**B3**,**C1**–**C3**,**D1**–**D3**,**E1**–**E3**) Immunofluorescence staining of DAPI, GFAP, and Map-2 of Aligned, Random, and SCI groups (scale bar = 500 μm). (**F**–**H**) Fluorescence intensity statistics of DAPI, GFAP, and Map-2 in Aligned, Random, SCI group. The data are presented as Mean ± SD; ** *p* < 0.01, *** *p* < 0.005; ns: no significant; data are representative of at least three independent experiments unless otherwise indicated.

One of the most crucial stages of neuronal regeneration following injury is the formation of new axons by newborn neurons. However, following injury, astrocytes accumulate at the site of injury and form scar tissue, which impedes the regeneration of axons. The astrocytes and nascent axons at the injury site were evaluated using GFAP and Map-2 markers (Figure 7A–E). The analysis was performed semi-quantitatively, considering the number of positive cells and the positive area, which were then statistically analyzed (Figure 7F–H). Upon examination of the MAP-2-positive cells, it was discovered that the Aligned group (30.16 ± 5.31) had the highest MAP-2 fluorescence intensity compared to the other two groups (Random: 19.76 ± 4.84; SCI: 14.02 ± 2.11). Interestingly, the neurons at both ends of the damaged area in this group exhibited the growth facing the same direction, indicating the possibility of a new neural pathway forming, which was not observed in the Random group. GFAP-positive cells were evident in the Random (28.87 ± 2.46) and SCI (24.13 ± 3.56) groups, except for the Aligned group (13.41 ± 2.31). The location of GFAP presence overlapped with Map-2, which is consistent with the notion that glial scarring hinders axonal regeneration. As depicted in the provided (Figure 7F–H), an analysis of the fluorescence intensity for DAPI, GFAP, and MAP-2 revealed a lack of significant variance between the Random and SCI groups. Contrarily, this variance was prominently observed within the Aligned group. These findings collectively suggest that the aligned MSCs@DSC/Gel scaffolds will facilitate positive outcomes in nerve regeneration and reconstruction of neural pathways.

To summarize, the MSCs@DSC/Gel can operate effectively in the rat SCI model to facilitate neuronal compensation and benefits following injury. The scaffolds can provide a favorable microenvironment at the injury site, encourage the restoration and regrowth of neural tissues, and improve behavioral function.

## 4. Discussion

Traditional pharmacotherapy and surgical interventions have demonstrated restricted efficacy, necessitating the exploration of alternative treatment modalities. Consequently, cell transplantation therapy and biomaterial implantation have emerged as subjects of extensive research in recent years [3,37], offering promising avenues for addressing the limitations of conventional therapeutic approaches [4,38]. Therefore, the microenvironment remodeling for reconnection of neuronal circuits is key to the viable treatments after SCI [39]. However, traditional biomaterial is insufficient to counteract the cell loss resulting from damage. Transplanted cells provide physical and nutritional support to the damaged tissues, but the inhibitory microenvironment compromises their immunomodulatory, proliferative, and differentiation potential [40,41,42]. In our study, we combine cell transplantation methodologies with biomaterial technology, culminating in developing aligned DSC nanofiber with MSCs. This approach is designed to optimize the regenerative capabilities of MSCs upon their transplantation into areas of injury. The structural alignment of nanofibers plays a pivotal role in enhancing the regenerative potential of MSCs, notably facilitating neuronal differentiation and axonal regeneration. Thus, it could promote the reconstruction of neural pathways and enhance motor function recovery after SCI.

Recent developments in tissue engineering have revealed that ECM shows promise as a material for repairing neural damage [20,43]. This is due to its exceptional biocompatibility and minimal immunogenicity, primarily attributed to its low DNA content and protein fractions [44,45]. In our study, we successfully decellularized the porcine spinal cord using physical, chemical, and biological methods. This process effectively eliminated most of the immunogenic components and significantly reduced immunogenicity. However, it retained essential proteins like GAG, which promotes neuronal development and neural protrusion growth; laminin, which regulates neuronal adhesion and growth and promotes central nervous system development; fibronectin, which regulates cell adhesion and migration; and collagen, an essential ECM component involved in nerve repair and regeneration. These ECM proteins play a crucial role in cellular behaviors like adhesion, proliferation, and migration, creating an optimal environment for neurotrophy and neuroprotection [23,24,46].

The regeneration of nerve tissue requires scaffolds that possess suitable mechanical properties and can release specific guiding cues [12,47,48]. Electrospun fibers have become a promising avenue for neural tissue regeneration [49]; however, the electrospinning process presents a significant challenge for the fabrication of ECM. To address this, gelatin, a widely used bioactive material in tissue engineering, was incorporated into the solution of DSC in this study to enhance the processability and improve the mechanical properties of scaffolds [35,50]. Nevertheless, forming a homogeneous spinning liquid from gelatin and DSC remains a significant challenge. HFIP, a common solvent, can break the hydrogen bonds in the collagen triple helix structure and weaken the hydrophobic interactions between collagen molecular chains in ECM and gelatin, which is favorable for creating a homogeneous spinning solution. In this study, HFIP was chosen as the co-solvent of DSC and Gel, and the ratio of the formulation was adjusted to obtain a homogeneous spinning solution. Through the manipulation of voltage, we were able to produce fibers of varying diameters. Our selection of a suitable preparation process was based on multiple factors, including diameter and uniformity. It has been demonstrated that well-oriented fibers are more effective in promoting the directional growth of MSCs and inducing axonal myelin regeneration. To achieve this, we utilized a higher-speed fiber receiver to generate nanofibers with a higher degree of orientation than the random group. Our study involved a detailed optimization process for the DSC/Gel electrostatic spinning technique to create oriented nanofiber membranes.

MSCs had potent differentiation capabilities and clinical safety profiles, positioning them as a preferred candidate in neuronal compensation [51]. Despite their potential, the therapeutic outcomes following MSCs transplantation in clinical scenarios have been modest. The phenomenon can be attributed to several factors, such as a low survival rate post-transplantation and the unregulated migration and differentiation of these cells in vivo. MSCs transplantation may promote tissue regeneration and repair through various mechanisms, including the guidance of cell migration, modulation of immune responses, and facilitation of neuronal differentiation for axonal and myelin regeneration [52,53]. Due to functionally oriented structures in spinal cord nerve tissue and axons, we could design aligned fibers to create physical signal pathways for MSCs to aid in nerve regeneration [54,55,56,57]. In our study, the nanofiber mat exhibited limited stability in aqueous environments, necessitating a cross-linking treatment to enhance its durability. The processed mat demonstrated negligible cytotoxicity, laying the groundwork for subsequent MSCs loading experiments. The co-culture involving MSCs revealed that the orientation of fibers significantly influences the directional growth of MSCs. This finding suggests that MSCs could be retained at the injury site, supporting and directing neuronal migration across the injury gap via the nanofibrous scaffold. Gene expression analyses indicated that the fiber scaffolds could modulate the immune response of MSCs. Moreover, the expression levels of neuron-associated genes were markedly elevated in MSCs cultured with the oriented fibers compared to those in a disordered arrangement. The aligned DSC/Gel structure could induce MSCs differentiation towards neuronal phenotypes in vitro, potentially mitigating tissue loss resulting from spinal cord injuries and secreting nerve growth factors to facilitate nerve regeneration. The employment of structured fiber scaffolds not only augments the neural differentiation of MSCs but also exerts control over stem cell migration. Consequently, the synergistic integration of structured fiber scaffolds with MSCs emerges as a promising and beneficial strategy for the comprehensive treatment of SCI.

In this work, we established a spinal cord half-truncation model to assess the recovery capacity of aligned and random nanofibers after SCI. The findings revealed that the component of DSC in the scaffold may facilitate a more favorable environment for MSCs to secrete neuroprotective molecules; in our study, the aligned group showed higher therapeutic than the other two groups. Tissue and cell damage may create an unfavorable microenvironment, such as glial scarring, further hindering nerve regeneration after SCI. This is supported by the high overlap between GFAP and MAP-2 distribution, as observed in our study. Therefore, creating a favorable microenvironment is crucial when dealing with spinal cord injuries to promote the formation of new neural pathways and enhance kinematic recovery. The results showed a noticeable increase in neurons at the injury site and restoration of limb motor function using aligned MSCs@DSC/Gel scaffolds. As a result, the MSCs@DSC/Gel fibers display promising potential as scaffolds for SCI.

## 5. Conclusions

In this study, we developed an aligned fiber using decellularization and electrospinning for SCI. The scaffold closely resembles the structural and functional properties of the native spinal cord. The aligned structure guided directional migration and neuronal differentiation of MSCs during in vitro culture. In a rat hemisection model, the therapeutic efficacy of MSCs@DSC/Gel was evaluated, and the results indicated that the aligned scaffolds had positive effects on tissue regeneration and motor function recovery. These findings reveal that aligned MSCs@DSC/Gel scaffolds could be promising options for treating SCI in a clinical setting.

## Figures and Tables

**Table 1:** Primer sequences for qRT-PCR.

Genes (for mRNA)	Sequence (5′–3′)
GAPDH	Forward: GCCTCGTCTCATAGACAAGATGGT
	Reverse: GAAGGCAGCCCTGGTAACC
CD163	Forward: TGGCCTCTGAGTTTAGGGTCT
	Reverse: CCCTTGGTGTCGAACCAGC
CD86	Forward: CTGGACTCTACGACTTCACAATG
	Reverse: AGTTGGCGATCACTGACAGTT
Tuj-1	Forward: TAGACCCCAGCGGCAACTAT
	Reverse: GTTCCAGGTTCCAAGTCCACC
Nestin	Forward: GGGCCACTCCCTTCTCTAGT
	Reverse: AAGATTCTTCCCCGACGCAA
Map-2	Forward: GCCAGCCTCGGAACAAACA
	Reverse: GCTCAGCGAATGAGGAAGGA
GFAP	Forward: GGGGCAAAAGCACCAAAGAAG
	Reverse: GGGACAACTTGTATTGTGAGCC

**Table 2:** Effect of precursor liquid on spinning.

	DSC/Gel	1:0	1:1	1:3	1:4
HFIP/H_2_O					
**1:0**		+, **	+, ***	++, ***	++, ***
**10:1**		+, *	++, **	+++, **	+++, **
**5:1**		++, *	+++, **	+++, ***	+++, ***
**1:1**		++, *	+++, **	+++, **	+++, **

The table includes symbols to represent solubility and filament-forming properties. The symbol +, ++, and +++ represent poor, good, and excellent solubility, respectively. The symbol *, **, and *** represent poor, good, and excellent filament-forming properties, respectively.

## Data Availability

The original contributions presented in the study are included in the article, further inquiries can be directed to the corresponding author.
